# The *Saccharina latissima* microbiome: Effects of region, season, and physiology

**DOI:** 10.3389/fmicb.2022.1050939

**Published:** 2023-01-06

**Authors:** Bertille Burgunter-Delamare, Sylvie Rousvoal, Erwan Legeay, Gwenn Tanguy, Stein Fredriksen, Catherine Boyen, Simon M. Dittami

**Affiliations:** ^1^CNRS, Sorbonne Université, Integrative Biology of Marine Models (LBI2M), Station Biologique de Roscoff, Roscoff, France; ^2^FR2424 Station Biologique de Roscoff, CNRS, Sorbonne Université, Roscoff, France; ^3^Department of Biosciences, University of Oslo, Oslo, Norway

**Keywords:** holobiont, brown macroalgae, natural population, metabarcoding, microbiota

## Abstract

**Introduction:**

*Saccharina latissima* is a canopy-forming species of brown algae and, as such, is considered an ecosystem engineer. Several populations of this alga are exploited worldwide, and a decrease in the abundance of *S. latissima* at its southern distributional range limits has been observed. Despite its economic and ecological interest, only a few data are available on the composition of microbiota associated with *S. latissima* and its role in algal physiologyn.

**Methods:**

We studied the whole bacterial community composition associated with *S. latissima* samples from three locations (Brittany, Helgoland, and Skagerrak) by 16S metabarcoding analyses at different scales: algal blade part, regions, season (at one site), and algal physiologic state.

**Results and Discussion:**

We have shown that the difference in bacterial composition is driven by factors of decreasing importance: (i) the algal tissues (apex/meristem), (ii) the geographical area, (iii) the seasons (at the Roscoff site), and (iv) the algal host’s condition (healthy vs. symptoms). Overall, *Alphaproteobacteria*, *Gammaproteobacteria*, and *Bacteroidia* dominated the general bacterial communities. Almost all individuals hosted bacteria of the genus *Granulosicoccus*, accounting for 12% of the total sequences, and eight additional core genera were identified. Our results also highlight a microbial signature characteristic for algae in poor health independent of the disease symptoms. Thus, our study provides a comprehensive overview of the *S. latissima* microbiome, forming a basis for understanding holobiont functioning.

## 1. Introduction

Brown macroalgae, particularly kelps (Laminariales), play essential ecosystem engineering roles in coastal temperate marine environments. Depending on the genus, they are distributed across the western or eastern temperate North Pacific, the Arctic, and North Atlantic Oceans (Bolton, 2010; Araújo et al., 2016). Kelps contribute to primary productivity and are habitat formers providing food and shelter to the local biodiversity (Schiel and Foster, 2006; Schiel and Lilley, 2007). In addition, species of kelps are important in many industries to produce alginates (Peteiro, 2018), human food, medicine (Smit, 2004), or food for abalone aquaculture (McHugh, 2003; Roussel et al., 2019).

*Saccharina latissima* (Linnaeus) C.E. Lane, C. Mayes, Druehl & G.W. Saunders is one of the dominant kelp-forming species of brown macroalgae in Europe. This perennial species (2–5 years) is widely distributed in the North Atlantic, North Pacific, and the Arctic (Neiva et al., 2018). Its tissue growth starts from the meristematic region at the base of the blade, with the older tissue being at the apex part. These older parts can undergo erosion due to senescence and host a higher bacterial diversity, as shown in previous research on other *Laminariales*, notably *Laminaria digitata* (Corre and Prieur, 1990), *Laminaria hyperborea* (Bengtsson et al., 2010), *Laminaria longicruris* (Laycock, 1974), *Laminaria pallida* (Mazure and Field, 1980), and *Laminaria setchellii* (Lemay et al., 2021).

In recent years, a decrease in the abundance of *S. latissima* at its southern range limits has been observed (Araújo et al., 2016; Smale, 2020). The exact processes driving this decline are not fully understood, but it is likely that changes in peak temperature associated with changes in the microbiota might be at least partially linked to this process, as is the case with corals (Bourne et al., 2008; Bosch and Miller, 2016; Peixoto et al., 2017).

Indeed, macroalgal functioning needs to be seen as the result of the interactions between the algal hosts and their associated microbiota, constituting a singular entity termed the algal holobiont (Egan et al., 2013). It has been shown that macroalgal health, fitness, pathogen resistance (Wiese et al., 2009), acclimation to a changing environment (Dittami et al., 2016), and metabolism (Burgunter-Delamare et al., 2020) are regulated and supported by bacterial partners (Goecke et al., 2010). Considering the biofilm composition and deciphering the interactions within the holobiont is thus essential to fully understand the biology of algae. Previous studies were carried out on microbiota of different kelp species like *L. digitata* (Ihua et al., 2020), *L. hyperborea* (Bengtsson et al., 2010), *L. religiosa* (Vairappan et al., 2001), and *L. setchellii* (Lemay et al., 2021), but little is known about the *S. latissima* microbiota. Notably, Staufenberger et al. (2008) analyzed the bacterial composition of *Saccharina* from two locations and seasons (Baltic and North Sea; January and April 2006) using denaturing gradient gel electrophoresis (DGGE) and 16S rRNA gene clone libraries. Later, Tourneroche et al. (2020) used 16S metabarcoding and FISH to decipher the bacterial microbiota of young tissues of *S. latissima* sampled in Scotland on one date.

In the present study, we compared the microbiota composition of young *S. latissima* samples from several locations in the Atlantic Ocean (Roscoff, Helgoland, and Skagerrak) by 16S metabarcoding analyses to test if the microbiota is specific to the area of origin, season, and algal blade part (apex/meristem). Lastly, we compared the microbiota composition of healthy thalli with those of thalli exhibiting one or several symptoms (holes, bleaching, twisted blades). This enabled us to identify a microbial signature characteristic of algae in poor health, regardless of the precise symptoms.

## 2. Material and methods

### 2.1. Biological material and environmental variables

*Saccharina latissima* were sampled at different sites and dates (Table 1). Briefly, samples were taken from three regions (Roscoff, Helgoland, and Skagerrak) at low tide (or diving when necessary at the Helgoland and Skagerrak sites). Please note, however, that the tidal differences at Roscoff are up to 9 m, whereas they reach 3 m at Helgoland and only 30 cm in the Skagerrak on the coast of Norway. Among young individuals (<1 m length), five healthy algae and five with physical symptoms (holes, bleaching, twisted blades) were selected for each sampling session. We focused on a general “symptoms” category rather than on a specific disease because it was impossible to find enough individuals with the same symptoms throughout the sampling sessions and sites. The algal material was immediately placed in sterile plastic bags and rapidly (<3 h) transported to the laboratory in a cooling box at *ca.* 4°C.

**Table 1:** Sampling dates and sites.

Location	Latitude, Longitude	Date	Time	Types of samples
Roscoff, Brittany, France	48°43′47.0 “N 4°00′17.1” W	23 January 2019 18 April 2019 31 July 2019 29 October 2019	At low tide mid-day for all	Healthy + Symptoms
Helgoland, North Sea, Germany	54°10′47.3 “N 7°54′59.4” E 54°11′27.1 “N 7°52′02.4” E	10 July 2019 11 July 2019	Low tide	Healthy + Symptoms
Skagerrak, Norway	58°15′15.5 “N 8°31′22.2” E 58°22′05.1 “N 8°44′04.7” E 58°05′39.3 “N 6°34′54.9” E	16 October 2018 18 October 2018 1 April 2019	n/a	Healthy samples No samples with symptoms

**Figure 1:**
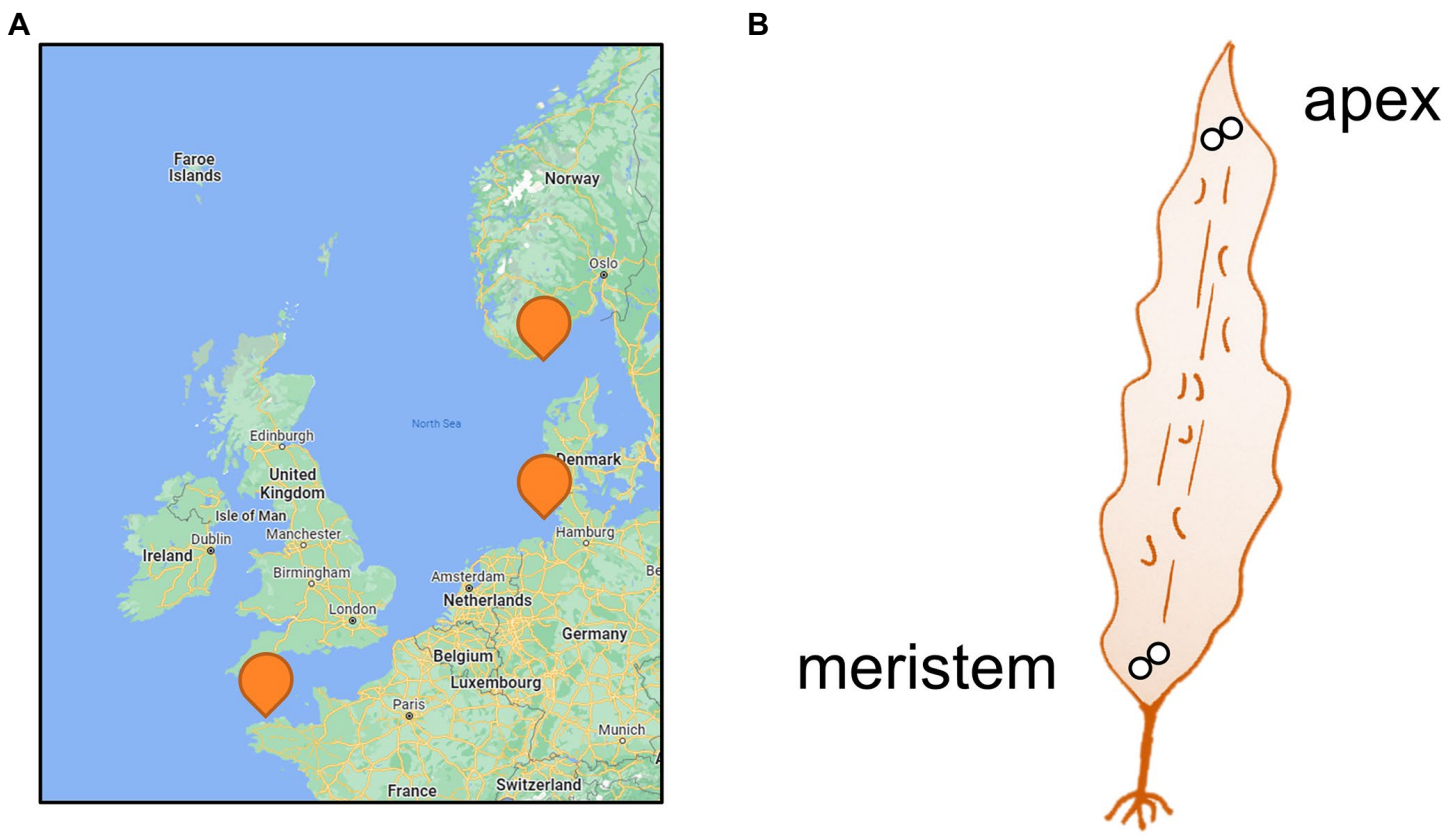
Sampling. **(A)** Map of sampling locations and **(B)** Sampled parts of the *Saccharina latissima* thalli (<1 m). Two discs (Ø2cm) were punched out in immediate proximity for each part of the blade and preserved in silica gel. Please refer to Table 1 for details on the sampling sites.

Two parts of the blades were sampled: the basal meristem and the tip (Figure 1). A disc with Ø2cm was punched out for each part of the blade and placed in a 15 ml Falcon tube containing 5 ml of clean silica gel (2-6 mm; VWR), following the protocol developed in our laboratory (Burgunter-Delamare et al., 2022). Tubes were stored at room temperature for up to 15 days before DNA extraction.

For the samples from Roscoff, corresponding environmental variables (temperature, salinity and ammonium, nitrites, nitrates, and phosphate concentrations) were obtained from the Service d’Observation en Milieu Littoral (SOMLIT) database (Astan point^1^; approximately 3.6 km North-East of the sampling point). They are available in Supplementary Figure S1. For the other sites, no corresponding environmental data was available.

### 2.2. DNA extraction

DNA extraction was carried out with the silica-gel stored samples, according to the protocol described by Bernard et al. (2017). Briefly, samples were freeze-dried, and ½ of a disk was ground using a Qiagen TissueLyser II bead beater (3 sessions, 45 s, 30 Hz, 3 mm stainless steel beads). Nucleic acids were then extracted using a 2% CTAB extraction buffer (100 mM Tris–HCl [pH 7.5], 1.5 M NaCl, 2% CTAB, 50 mM EDTA [pH 8], 50 mM DTT; shaker 250 rpm at room temperature). Supernatants were purified with one volume of chloroform/isoamyl alcohol (24:1) followed by 15 min centrifugation at 10000 rpm (16°C). The upper phase was transferred to a new tube, and ethanol (0.3 vol) was added drop by drop until polysaccharide precipitation was visible, followed by a second chloroform/isoamyl alcohol extraction and recovery of the aqueous phase. The pre-purified DNA was purified using Nucleospin plant II columns (Macherey-Nagel, Germany) according to the manufacturer’s instructions. Finally, DNA was eluted in 50 μl of elution buffer (Macherey-Nagel). Blank extractions were also performed. These extracts were used to identify potential contaminations introduced during the extraction and downstream processing of the samples.

### 2.3. 16S Metabarcoding

The bacterial community composition associated with algal cultures was determined by 16S metabarcoding. A mock community comprising a mix of DNA from 26 cultivated bacterial strains (Thomas et al., 2019) and negative control were run and treated in parallel to the DNA extracts. For all of these samples, the V3 and V4 regions of the 16S rDNA gene were amplified using the NOCHL primers including Illumina adapters (Thomas et al., 2019), to avoid plastid DNA amplification. Then a standard Illumina protocol for metabarcoding (Illumina, 2013) was run using the Q5® High-Fidelity PCR Kit (New England BioLabs, MA, USA), the AMPure XP for PCR Purification Kit (Beckman Coulter, Brea, CA, USA), and the Nextera XT DNA Library Preparation Kit (Illumina, San Diego, CA, USA). Libraries were quantified with a Quantifluor® ds DNA System (Promega, WI, USA), and mean fragment size was determined using a LabChip® GX Touch™ (Perkin Elmer, MA, USA). An equimolar pool of all samples was generated at a concentration of 4 nM, diluted to 3 pM, spiked with 10% PhiX (Illumina), and sequenced on an Illumina MiSeq sequencer at the Genomer platform (Station Biologique de Roscoff) using a MiSeq v3 kit (2x300bp, paired-end). Raw Illumina reads were deposited at the European Nucleotide Archive under project accession number PRJEB47035.

### 2.4. Analyses

Sequence analysis was performed using the DADA2 1.14.0 package (Callahan et al., 2016) on R 3.6.2 following the protocol established by Benjamin Callahan^2^. Sequences were filtered, allowing for a maximum of 2 expected errors and reducing the read length to 291 bp for forward reads and 265 bp for reverse reads. An amplicon sequence variant (ASV) table was constructed, and chimeras were removed. The taxonomy of the remaining ASVs was assigned using the Silva_SEED 138 database. The resulting abundance table and taxonomic classification were analyzed using Phyloseq 1.30.0 (McMurdie and Holmes, 2013). ASVs that were more abundant in the blank samples than in the algal samples, organellar and eukaryote reads, rare ASVs (<0.01% of total reads), and samples with less than 7,688 remaining reads were removed. Non-Metric Multidimensional Scaling analyses (NMDS) were carried out using the Bray-Curtis dissimilarities and the vegan R package version 2.6–2. We tested for factor effects on community dissimilarity using permutational analysis of variance (PERMANOVA; Anderson, 2001) using the adonis2 function (vegan R package). The most important factor separating the samples in the NMDS was then further explored. The Shannon H diversity index was calculated using Past version 4.02 (Hammer et al., 2001). The normality of the input data was verified with a Shapiro–Wilk test. If they followed a normal distribution, the variance homogeneity was tested by a Levene test, followed by a t-test. If the data were not normal, a Kruskal Wallis test or a Mann–Whitney test was performed. Statistical analysis of differential abundance was performed at the genus and phylum level using ANCOM-BC version 1.4.0 (Lin and Peddada, 2020) with default parameters. Binomial tests followed by a Benjamini and Hochberg (BH) correction (Benjamini and Hochberg, 1995) were carried out to determine the overrepresented genera among the ASVs identified by ANCOM-BC. Then, the factor in question was eliminated from the dataset if possible (grouping of apex and meristem sample, focus on specific region), and the analyses were repeated to determine the next factor. Even if there is no consistent definition of the core and threshold values can range from 50% to 100% (Shade and Handelsman, 2012; Risely, 2020), we choose to determine the bacterial core at the genus level and define it as genera present in 90% of replicates for each algal part, season, and location. This is a rather stringent definition excluding, for instance, bacteria that are associated with the algal hosts only at particular ages or seasons.

## 3. Results

### 3.1. General taxonomy

16S metabarcoding analyses were carried out for all control and algal samples. A total of 4,028,372 raw sequences were generated and, after filtering, assembled into 1,658,746 merged contigs. The taxonomic assignation of mock samples was consistent with the mock composition, and a total of 18,028 ASVs were identified in the dataset. The sequences obtained corresponded predominantly to *Alphaproteobacteria* (34.1% of total reads), followed by *Gammaproteobacteria* (29.5% of total reads) and *Bacteroidota* (26% of total reads).

### 3.2. Comparison of apex and meristem samples

**Figure 2:**
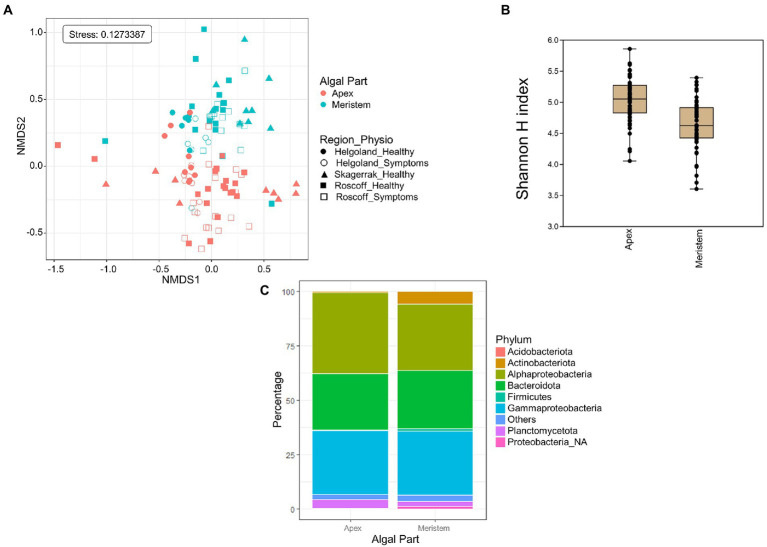
Algal blade part analysis. **(A)** NMDS analysis of the microbiome composition. Results show a clear separation of the apex and meristem samples (PERMANOVA, *p* = 0.001). **(B)** Box plot of alpha-diversity (Shannon H index) across different sample types calculated at the ASV level. Error bars correspond to the range; *n* = 111; mean alpha-diversity differs significantly between conditions (*t*-test, *t* = 5.47, *p* < 0.0001; **C)** Comparison of microbiome composition between apex and meristem samples at the phylum level. Bars show the relative abundance of 16S rRNA gene metabarcoding sequences; all phyla were detected in all sample types, and only their relative abundance varied. *Proteobacteria_NA*: unclassified as *Proteobacteria*.

Global NMDS analysis of all samples demonstrated a clear separation between the apex and meristem samples (PERMANOVA *p* = 0.001; Figure 2A). We also observed a significant interaction between the algal part and the region factors (PERMANOVA *p = 0.001*), as several apex samples from Helgoland group with meristem samples, while one meristem sample from Roscoff grouped with the apex sample. Overall, alpha diversity, as calculated using the Shannon H index (Figure 2B), was higher in apex samples than in meristem samples (*p*-value < 0.0001). Several phyla were found to differ significantly in relative abundance between the apex and meristem samples. The *Actinobacteriota*, *Firmicutes*, and unclassified *Proteobacteria* (*p* < 0.0001) were found in higher relative abundance in the meristem samples. The *Alphaproteobacteria* (*p* = 0.00016) class, and the *Bacteroidota* (*p* = 0.0037), and *Planctomycetota* (*p* = 0.004) phyla were relatively more abundant in the apex samples (Figure 2C). ANCOM-BC analyses revealed a total of 122 ASVs to differ significantly (adjusted (*p*-value < 0.05) in relative abundance between the apex and meristem samples (28 ASVs were more abundant in apex and 94 in meristem samples; Supplementary Table S1). The taxonomic groups overrepresented (adjusted (*p*-value < 0.05; BH correction) among these significant ASVs are shown in Table 2: one genus was significantly overrepresented in the apex samples (*Ki89A_clade*, 39%) and seven in the meristem samples (four of them belong to the *Gammaproteobacteria*; Table 2). The bacterial core in the apex and meristem samples comprises the four genera *Granulosicoccus*, *Litorimonas*, *Hellea,* and *Blastopirellula,* accounting for 32% of the total reads for all samples. Five additional genera were systematically present in the apical part: *Algitalea*, *Arenicella*, *Portibacter*, *Tenacibaculum*, and *Bdellovibrio* and accounted for 15% of the total reads.

**Table 2:** Taxonomic affiliations of the ASVs characteristic for apex and meristem samples, compared with their occurrence in the entire dataset.

Taxa	APEX				MERISTEM				Entire dataset		
	Number of over-expressed ASVs	Total number of over-expressed ASVs	Ratio	*p*-value	Number of over-expressed ASVs	Total number of over-expressed ASVs	Ratio	*p*-value	Number of ASVs	Total number of ASVs	Ratio
*KI89A_clade*	11	28	0.393	<0.00001 ***	0	94	0.0000	n.c.	100	16,689	0.0060
*Litorimonas*	3	28	0.107	0.01132	13	94	0.1383	<0.00001 ***	530	16,689	0.0318
*Maribacter*	0	28	0	n.c.	15	94	0.1596	<0.00001 ***	89	16,689	0.0053
*Octadecabacter*	0	28	0	n.c.	5	94	0.0532	<0.00001 ***	73	16,689	0.0044
*Sva0996_marine_group*	0	28	0	n.c.	14	94	0.1489	<0.00001 ***	115	16,689	0.0069
*Proteobacteria_NA*	0	28	0	n.c.	4	94	0.0426	0.00004 ***	66	16,689	0.0040
*Granulosicoccus*	3	28	0.107	0.05723	14	94	0.1489	0.00012 ***	878	16,689	0.0526
*Rickettsiales_NA*	0	28	0	n.c.	1	94	0.0106	0.0053 ***	19	16,689	0.0011

n.c.: not calculated. *p*-values are the uncorrected results of a binomial test, and ***, **, and *indicate that these *p*-values were significant (i.e., the genus is significantly overrepresented among the genera regulated by tissue type) even after a Benjamini and Hochberg correction (corrected *p* < 0.001, <0.01, and <0.05, respectively).

### 3.3. Comparison of regions

**Figure 3:**
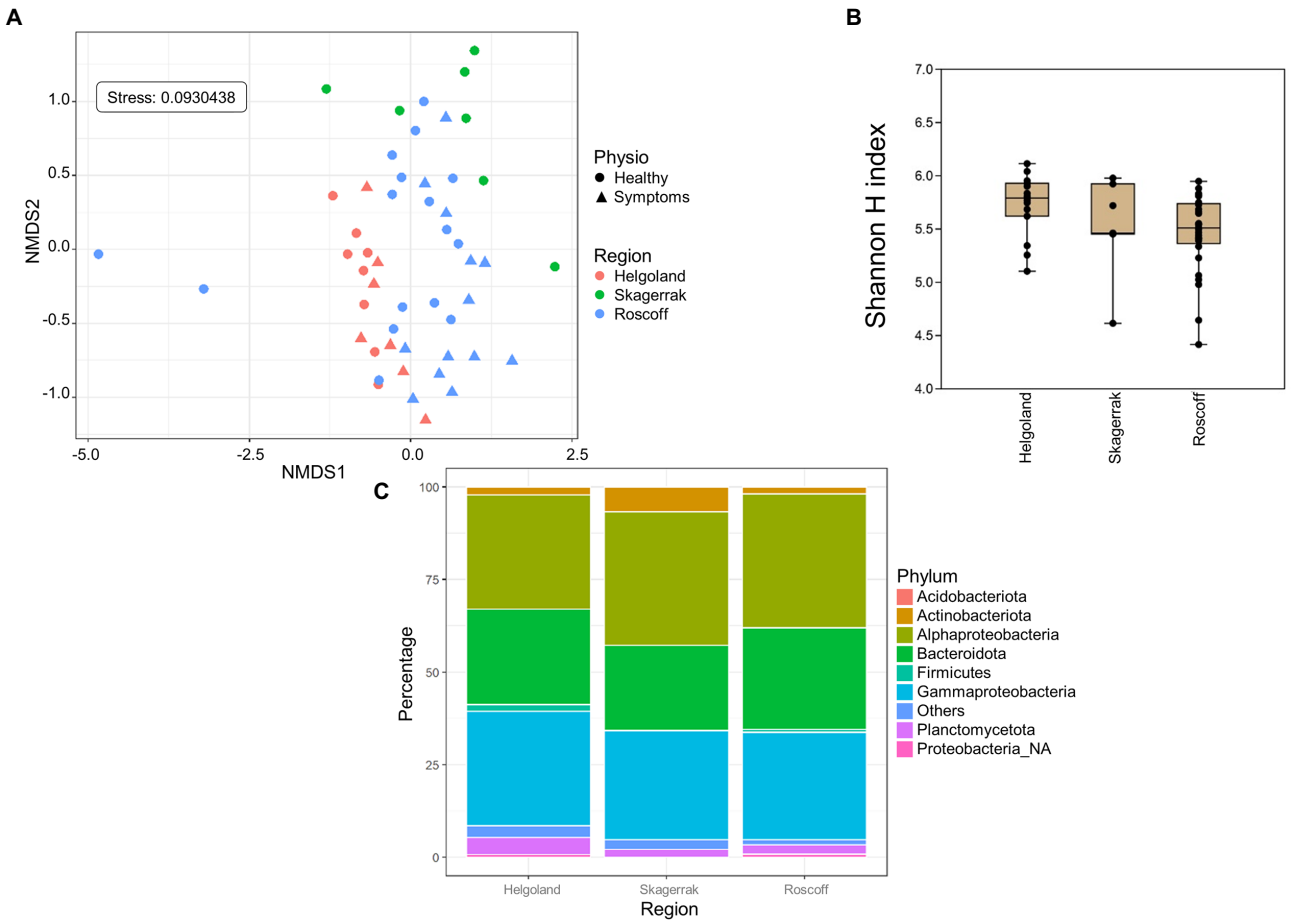
Region analysis. **(A)** NMDS analysis of the microbiome composition. Results show a clear separation of the samples according to their origin (PERMANOVA, *p* = 0.001). **(B)** Box plot of alpha-diversity (Shannon H index) across different sample types calculated at the ASV level. Error bars correspond to the range; *n* = 51; conditions do not differ significantly (Kruskal–Wallis test, *p* > 0.05). **(C)** Microbiome composition of samples from Helgoland, Skagerrak, and Roscoff at the phylum level. Bars show the relative abundance of 16S rRNA gene metabarcoding sequences; all phyla were detected in all sample types, and only their relative abundance varied. *Proteobacteria_NA*: unclassified Proteobacteria.

For the following analyses, reads from the apex and meristem samples of the same alga were pooled as individuals to remove the apex/meristem effect. On the NMDS plot, the samples are now grouped according to their region of origin (PERMANOVA *p* = 0.001; Figure 3A). The alpha diversity did not differ significantly between the regions (Figure 3B). However, at the phylum level, the *Firmicutes* and unclassified *Proteobacteria* were underrepresented in the Norwegian samples compared to Roscoff (ANCOM-BC, *p* = 0.013 and *p* < 0.0001) and Helgoland (ANCOM-BC, *p* = 0.004 and *p* < 0.0001; Figure 3C). *Bacteroidota* and *Alphaproteobacteria* exhibited significantly higher relative abundance in Roscoff than in Helgoland (ANCOM-BC, *p* = 0.003 for both phyla). At the ASV level, 234 ASVs were represented in higher proportions in the Roscoff samples, 243 in the Helgoland samples, and 18 in the samples from Skagerrak (Supplementary Table S1). The taxonomic affiliation of significantly over-expressed ASVs (adjusted *p*-value < 0.05; BH correction) is shown in Table 3. Twelve genera were significantly overrepresented in Helgoland samples (including *Alphaprotebacteria* 28.4% of ASVs and *Bacteroidota* 13.5% of ASVs), four genera in the Norwegian samples (including *Rhizobiaceae_NA*; 50% of ASVs), and 11 genera in Roscoff samples (nine of them belong to the *Proteobacteria*; Table 3).

**Table 3:** Taxonomic affiliations of the ASVs characteristic for the Roscoff, Helgoland, or Skagerrak samples, compared with their occurrence in the entire dataset.

Taxa	Roscoff				Helgoland				Skagerrak				Entire dataset		
	Number of over-expressed ASVs	Total number of over-expressed ASVs	Ratio	*p*-value	Number of over-expressed ASVs	Total number of over-expressed ASVs	Ratio	*p*-value	Number of over-expressed ASVs	Total number of over-expressed ASVs	Ratio	*p*-value	Number of ASVs	Total number of ASVs	Ratio
*KI89A_clade*	19	234	0.0812	<0.00001***	0	243	0	n.c.	2	18	0.111	0.00016 ***	100	16,689	0.0060
*Litorimonas*	23	234	0.0983	<0.00001***	46	243	0.189	<0.00001 ***	0	18	0	n.c.	530	16,689	0.0318
*Maribacter*	14	234	0.0598	<0.00001***	0	243	0	n.c.	0	18	0	n.c.	89	16,689	0.00533
*Octadecabacter*	12	234	0.0513	<0.00001***	0	243	0	n.c.	0	18	0	n.c.	73	16,689	0.00437
*Reichenbachiella*	19	234	0.0812	<0.00001***	0	243	0	n.c.	0	18	0	n.c.	258	16,689	0.0155
*Tateyamaria*	7	234	0.0299	<0.00001 ***	0	243	0	n.c.	0	18	0	n.c.	49	16,689	0.00294
*Yoonia-Loktanella*	10	234	0.0427	<0.00001 ***	0	243	0	n.c.	0	18	0	n.c.	91	16,689	0.00545
*Algimonas*	4	234	0.0171	0.00002 ***	0	243	0	n.c.	0	18	0	n.c.	22	16,689	0.00131
*Granulosicoccus*	23	234	0.0983	0.00150**	17	243	0.070	0.0922	0	18	0	n.c.	878	16,689	0.0526
*Proteobacteria_NA*	4	234	0.0171	0.00257 **	0	243	0	n.c.	0	18	0	n.c.	66	16,689	0.00395
*Hyphomonadaceae_NA*	11	234	0.0470	0.0064**	0	243	0	n.c.	0	18	0	n.c.	369	16,689	0.0221
*Algitalea*	8	234	0.0342	0.0871	17	243	0.070	0.00001***	0	18	0	n.c.	378	16,689	0.0226
*Blastopirellula*	2	234	0.0085	0.687	14	243	0.0576	0.00001***	0	18	0	n.c.	252	16,689	0.0151
*Thalassotalea*	0	234	0	n.c.	6	243	0.0247	0.00040***	0	18	0	n.c.	90	16,689	0.00539
*Lewinella*	0	234	0	n.c.	7	243	0.0288	0.00084 ***	0	18	0	n.c.	133	16,689	0.00797
*Rhodobacteraceae_NA*	1	234	0.0043	0.999	19	243	0.0782	0.00197 **	1	18	0.0556	0.160	666	16,689	0.0399
*Paraglaciecola*	1	234	0.0043	0.0915	3	243	0.0123	0.00204 **	0	18	0	n.c.	36	16,689	0.00216
*Sporolactobacillus*	1	234	0.0043	0.123	3	243	0.0123	0.00382 **	0	18	0	n.c.	43	16,689	0.00258
*Arenicella*	5	234	0.0214	0.860	17	243	0.070	0.00399 **	3	18	0.167	0.00364 **	611	16,689	0.0366
*Tenacibaculum*	6	234	0.0256	0.0866	9	243	0.0370	0.00661 **	0	18	0	n.c.	269	16,689	0.0161
*Sva0996_marine_group*	2	234	0.0085	0.220	5	243	0.0206	0.00723 **	0	18	0	n.c.	115	16,689	0.00689
*Robiginitomaculum*	2	234	0.0085	0.115	4	243	0.0165	0.00817 **	0	18	0	n.c.	84	16,689	0.00503
*Rhizobiaceae_NA*	0	234	0	n.c.	0	243	0	n.c.	9	18	0.5	<0.00001 ***	97	16,689	0.00581
*Zobellia*	0	234	0	n.c.	0	243	0	n.c.	1	18	0.0556	0.00178 **	58	16,689	0.00348

n.c.: not calculated. *p*-values are the uncorrected results of a binomial test, and ***, **, and *indicate that these *p*-values were significant (i.e., the genus is significantly overrepresented among the genera regulated by tissue type) even after a Benjamini and Hochberg correction (corrected *p* < 0.001, <0.01, and < 0.05, respectively).

### 3.4. Seasonality

**Figure 4:**
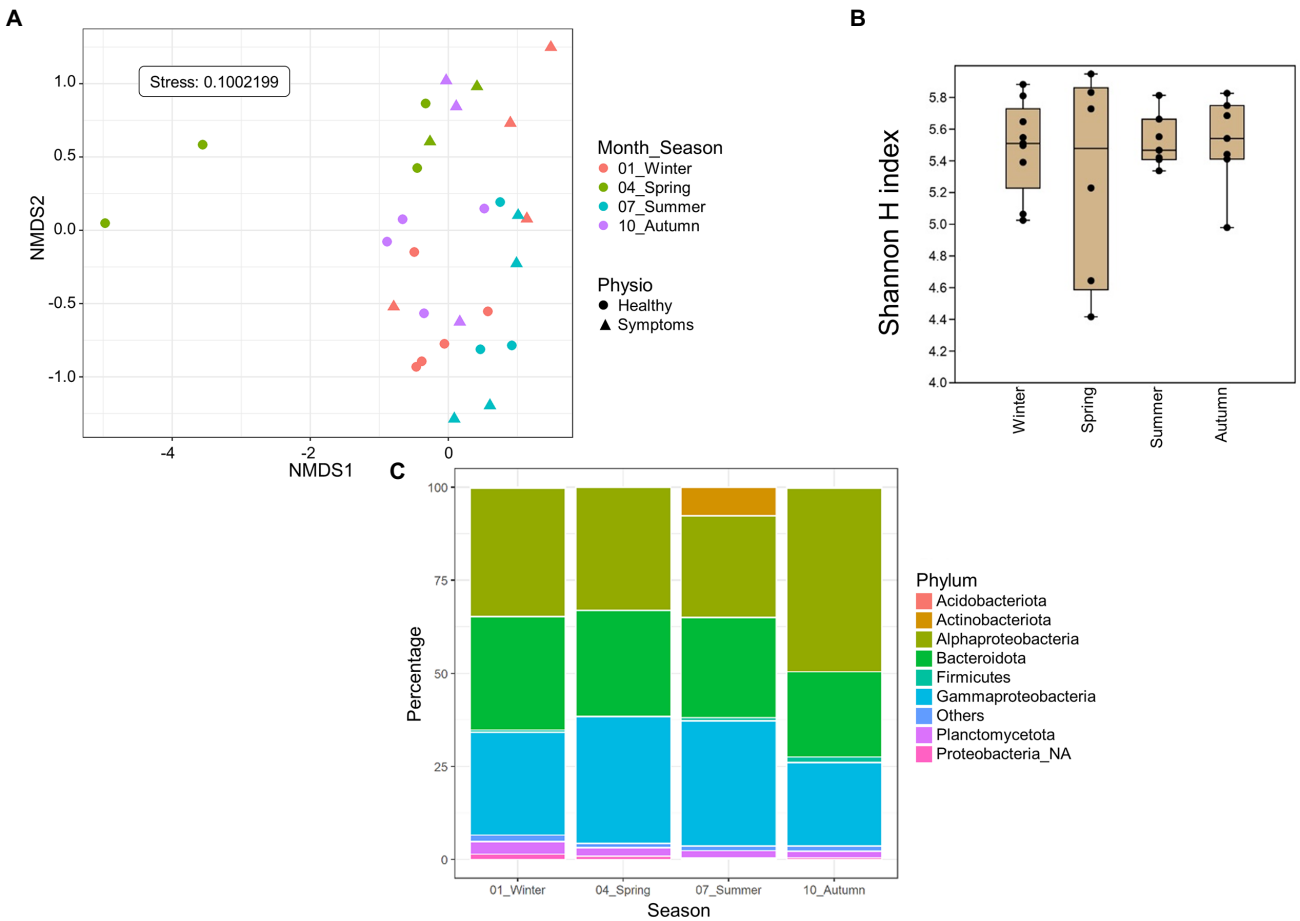
Season analyses. Only Roscoff samples were used to assess the impact of the season. **(A)** NMDS analysis of the microbiome composition. Results show a separation of the samples depending on the sampling season (PERMANOVA, *p* = 0.001). **(B)** Box plot of alpha-diversity (Shannon H index) across different sample types calculated at the ASV level. Error bars correspond to the range; n = 30; conditions do not differ significantly (Kruskal–Wallis test, *p > 0.05*). **(C)** Seasonal differences in microbiome composition in Roscoff at the phylum level. Bars show the relative abundance of 16S rRNA gene metabarcoding sequences; all phyla were detected in all sample types, and only their relative abundance varied. *Proteobacteria_NA*: unclassified Proteobacteria.

Only Roscoff samples (apex and meristem reads of the same alga pooled as individuals) were used to assess the impact of season on the microbiome because these were the only samples with four sampling points from different seasons available. The NMDS analysis shows a separation between different seasons’ samples. The spring and summer samples formed separate clusters, whereas the autumn and winter samples overlapped in an intermediate position between the two spring and summer samples (PERMANOVA *p = 0.001*; Figure 4A), even if the alpha diversity (Figure 4B) did not differ significantly between the seasons. *Actinobacteria* were exclusively found in summer. *Firmicutes* (ANCOM-BC, *p* = 0.032) were more abundant in autumn samples than in spring samples (ANCOM-BC, *p* = 0.032). *Alphaproteobacteria* were significantly more abundant in autumn than summer (ANCOM-BC, *p* = 0.0017) and winter (ANCOM-BC, ANCOM-BC *p* = 0.014). *Gammaproteobacteria* were significantly more abundant in spring than in autumn (ANCOM-BC, *p* = 0.032) and winter (*p* = 0.022; Figure 4C). ANCOM-BC analyses revealed 422 ASVs with higher relative abundance in one or several seasons. 126 ASVs were most abundant in winter samples, 85 ASVs in spring samples, 95 ASVs in summer samples, and 115 ASVs in autumn samples (Supplementary Table S1). The taxa significantly overexpressed among these ASVs (adjusted *p*-value < 0.05; BH correction) are shown in Table 4. Most ASVs with higher relative abundance belonged to the *Alphaproteobacteria* in the spring (*Litorimonas, Robiginitomaculum* and *Yoonia-Loktanella*; 27.1% of ASVs), and autumn samples (*Algitalea*, *Octadecabacter*, *Tateyamaria*, *Litorimonas*, and *Yoonia-Loktanella*; 37.4% of ASVs). In winter the over-represented ASVs belong to the *Gammaproteobacteria* (three genera; 28.6% of ASVs), *Alphaproteobacteria* (*Octadecabacter*, *Litoreibacter* and *Yoonia-Loktanella*; 17.5% of ASVs), and *Bacteroidota* (one genus; 8% of ASVs). In autumn, the *Cytophagales*_NA and *Algitalea* genera were also over-represented (*Bacteroidota*, representing 14.8% of ASVs). And in summer, eight genera were over-represented, and 33% of ASVs belonged to the *Gammaproteobacteria*. Also, the Sva0996_marine_group (*Actinobacteria;* 12%) was overrepresented only in these samples.

**Table 4:** Taxonomic affiliations of the ASVs characteristic for each season from Roscoff samples, compared with their occurrence in the entire dataset.

Taxa	Winter				Spring				Summer				Autumn				Entire dataset		
	Number of over-expressed ASVs	Total number of over-expressed ASVs	Ratio	*p*-value	Number of over-expressed ASVs	Total number of over-expressed ASVs	Ratio	*p*-value	Number of over-expressed ASVs	Total number of over-expressed ASVs	Ratio	*p*-value	Number of over-expressed ASVs	Total number of over-expressed ASVs	Ratio	*p*-value	Number of ASVs	Total number of ASVs	Ratio
*Gammaproteobacteria_NA*	20	126	0.159	<0.00001 ***	5	85	0.0588	0.168	3	95	0.0316	0.600	2	115	0.0174	0.883	729	16,689	0.0437
*Octadecabacter*	17	126	0.135	<0.00001 ***	1	85	0.0118	0.0538	0	95	0	n.c.	3	115	0.0261	0.00172 **	73	16,689	0.00437
*Reichenbachiella*	10	126	0.0794	<0.00001 ***	0	85	0	n.c.	0	95	0	n.c.	0	115	0	n.c.	258	16,689	0.0155
*Yoonia-Loktanella*	5	126	0.0397	0.00007 ***	5	85	0.0588	0.00001 ***	0	95	0	n.c.	4	115	0.0348	0.00045 ***	91	16,689	0.00545
*Escherichia/Shigella*	2	126	0.0159	0.00043 ***	2	85	0.0236	0.00014 ***	0	95	0	n.c.	0	115	0	n.c.	19	16,689	0.00114
*Litoreibacter*	2	126	0.0159	0.00248 **	1	85	0.0118	0.0140	0	95	0	n.c.	0	115	0	n.c.	35	16,689	0.00210
*Granulosicoccus*	14	126	0.111	0.00266 **	2	85	0.0236	0.831	12	95	0.126	0.00143 **	4	115	0.0348	0.729	878	16,689	0.0526
*Litorimonas*	4	126	0.0317	0.371	15	85	0.176	<0.00001 ***	7	95	0.0737	0.0109	26	115	0.226	<0.00001 ***	530	16,689	0.0318
*Flavobacteriaceae_NA*	3	126	0.0238	0.311	7	85	0.0824	0.00065 ***	0	95	0	n.c.	1	115	0.0087	0.730	373	16,689	0.0224
*Paraglaciecola*	0	126	0	n.c.	2	85	0.0236	0.00087 ***	0	95	0	n.c.	0	115	0	n.c.	36	16,689	0.00216
*Robiginitomaculum*	1	126	0.00794	0.133	3	85	0.0353	0.00094 ***	0	95	0	n.c.	1	115	0.0087	0.115	84	16,689	0.00503
*Portibacter*	1	126	0.00794	0.508	5	85	0.0588	0.00106 **	0	95	0	n.c.	0	115	0	n.c.	225	16,689	0.0135
*Tenacibaculum*	0	126	0	n.c.	5	85	0.0588	0.00259 **	1	95	0.0105	0.454	1	115	0.0087	0.555	269	16,689	0.0161
*Arenicella*	8	126	0.0635	0.0426	4	85	0.0471	0.201	14	95	0.147	<0.00001 ***	5	115	0.0435	0.246	611	16,689	0.0366
*Sva0996_marine_group*	0	126	0	n.c.	0	85	0	n.c.	12	95	0.126	<0.00001 ***	0	115	0	n.c.	115	16,689	0.00689
*KI89A_clade*	0	126	0	n.c.	0	85	0	n.c.	5	95	0.0526	0.00003 ***	0	115	0	n.c.	100	16,689	0.006
*Dokdonia*	2	126	0.0159	0.500	1	85	0.0118	0.539	9	95	0.0947	0.00004 ***	2	115	0.0174	0.440	353	16,689	0.0212
*Hyphomonadaceae_NA*	5	126	0.0397	0.0618	0	85	0	n.c.	8	95	0.0842	0.00027 ***	6	115	0.0522	0.0143	369	16,689	0.0221
*Algimonas*	0	126	0	n.c.	0	85	0	n.c.	2	95	0.0211	0.00029 ***	2	115	0.0174	0.00051 ***	22	16,689	0.00132
*Planctomycetales_NA*	0	126	0	n.c.	0	85	0	n.c.	1	95	0.0105	0.00654 **	0	115	0	n.c.	21	16,689	0.00126
*Tateyamaria*	1	126	0.00794	0.0534	1	85	0.0118	0.0262	1	95	0.0105	0.0321	8	115	0.0696	<0.00001 ***	49	16,689	0.00294
*Algitalea*	4	126	0.0317	0.159	0	85	0	n.c.	0	95	0	n.c.	15	115	0.130	<0.00001 ***	378	16,689	0.0226
*Cytophagales_NA*	0	126	0	n.c.	0	85	0	n.c.	0	95	0	n.c.	2	115	0.0174	0.00387 **	45	16,689	0.0027

n.c.: not calculated. *p*-values are the uncorrected results of a binomial test, and ***, **, and *indicate that these *p*-values were significant (i.e., the genus is significantly overrepresented among the genera regulated by tissue type) even after a Benjamini and Hochberg correction (corrected *p* < 0.001, <0.01, and < 0.05, respectively).

### 3.5. Comparison healthy/symptoms

**Figure 5:**
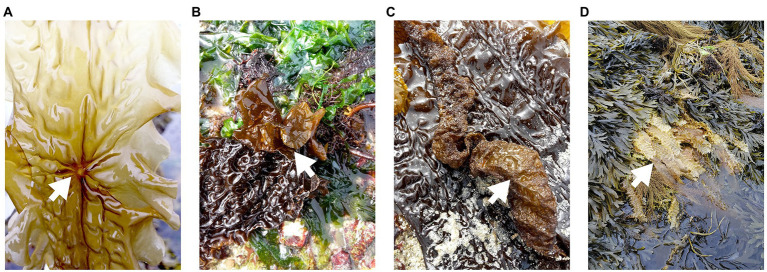
Examples of symptoms observed on “diseased” *S. latissima* individuals. **(A)** hole, **(B)** twisted blade, **(C)** bubbling in blade, and **(D)** bleaching.

**Figure 6:**
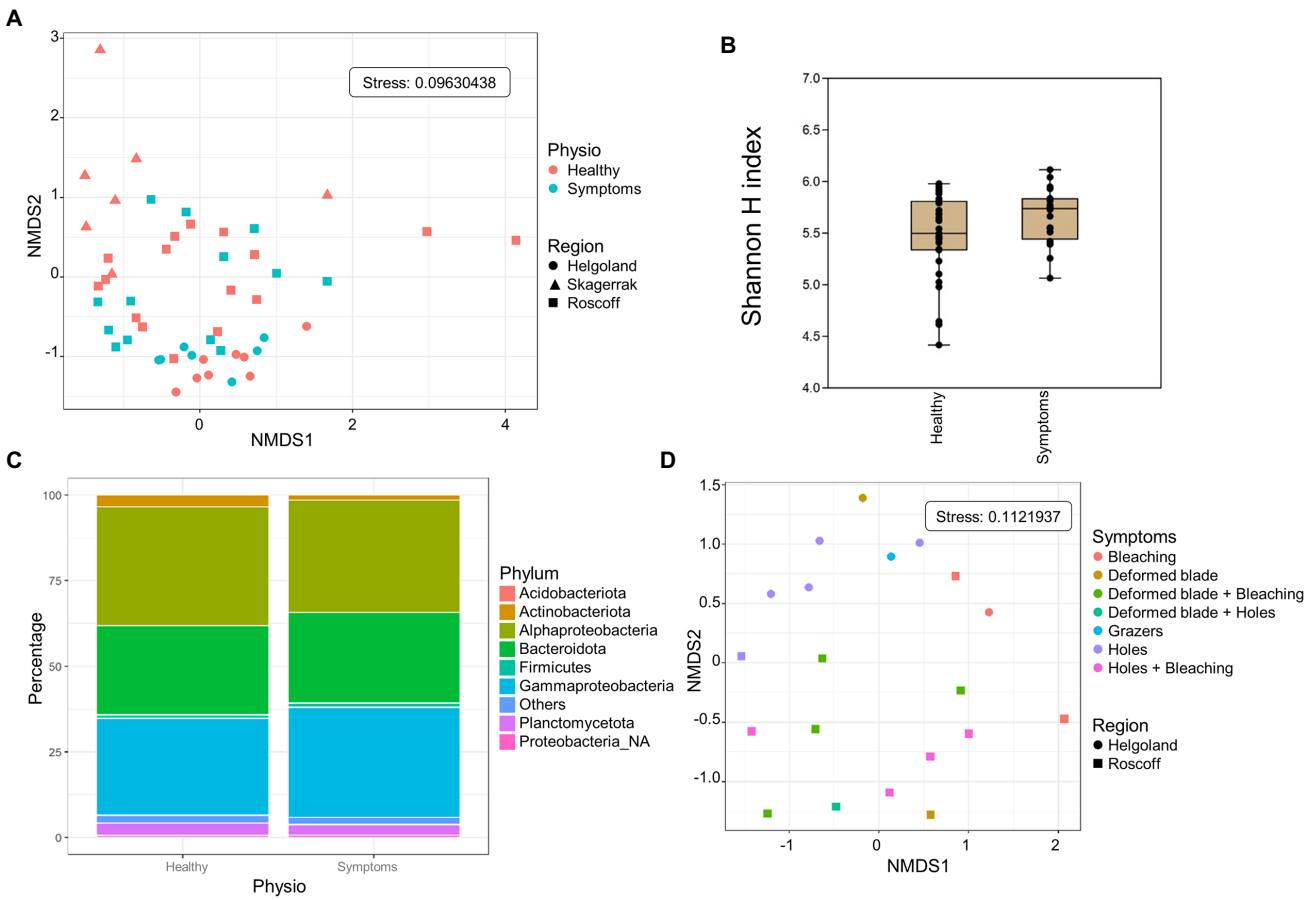
Analysis of samples with symptoms from Roscoff and Helgoland. **(A)** NMDS analysis of the microbiome composition. Results do not show a separation of the healthy and symptomatic samples (PERMANOVA *p* > 0.05). **(B)** Box plot of alpha-diversity (Shannon H index) across different sample types calculated at the ASV level. Error bars correspond to the range; n = 51, conditions do not differ significantly (Mann–Whitney pairwise test, *p* > 0.05). **(C)** Microbiome composition of healthy and symptoms samples at the phylum level. Bars show the relative abundance of 16S rRNA gene metabarcoding sequences; all phyla were detected in all sample types, and only their relative abundance varied. *Proteobacteria_NA*: unclassified Proteobacteria. **(D)** NMDS analysis of the microbiome composition depending on the symptoms.

Both healthy samples and samples with symptoms were found only in Roscoff and Helgoland, and the symptoms were diverse: holes, twisted blade, bubbling in the blade and bleaching (Figure 5). The NMDS shows no separation between the healthy individuals and those with symptoms (PERMANOVA, *p* > 0.05; Figure 6A), and the Shannon H index indicated no alpha diversity differences (*p* > 0.05; Figure 6B). No phyla significantly and systematically differed between healthy algae and algae with symptoms (Figure 6C). This observation also remains true when we distinguish samples between the different types of symptoms (Figure 6D), and the samples are still separated depending on the region. However, ANCOM-BC analyses revealed 9 ASVs that were characteristic in either of the groups: four ASVs were more abundant in samples with symptoms (*Alteromonadaceae_NA, Octadecabacter* sp., *Tenacibaculum* sp., and *Yoonia-Loktanella* sp.) and five that were more abundant in the healthy ones (*Escherichia/Shigella* sp., *Granulosicoccus* sp., *KI89A_clade*, *Rhodobacteraceae_NA*, *Zobellia* sp.; Supplementary Table S1).

## 4. Discussion

Knowledge of the bacterial communities within holobionts is key to understanding the dynamics of these systems. Here we studied the diversity and composition of bacterial communities of *S. latissima* by 16S metabarcoding analysis. The impacts of several factors on bacterial communities were examined: algal blade part, origin of the host, season, and host condition.

### 4.1. The blade part is the primary driver of samples separation

Distinct bacterial communities were demonstrated to be associated with different parts of *S. latissima* by 16S metabarcoding, and this is the primary factor of separation regardless of region, season, and physiology. Staufenberger et al. (2008) found the same dynamics when working on several *S. latissima* tissue from the Baltic Sea and the North Sea, sampled in winter and spring.

*Saccharina latissima’s* type of growth can explain this difference in bacterial communities. *Saccharina latissima* is a short-lived but perennial species, and growth occurs mainly in the meristem region. From there, the proliferating cells form the thallus. Young algae only have short blades and no access to the surrounding sediment as they only stand upright in the water column. As the thallus grows, it becomes heavier, and the apex finally bends and touches the ground (Kain, 1979; Lüning, 1991). Water currents move the old blade, resulting in access to nearby substrates and a broader environment. Mechanical stress also occurs in this part, which becomes vulnerable to bacterial decomposition, offering new ecological niches for different bacteria (Bengtsson and Øvreås, 2010). Therefore, the younger meristem tissues are typically less colonized by bacteria and exhibit lower bacterial diversity, as previously found (Staufenberger et al., 2008; Goecke et al., 2010; Ihua et al., 2020; Lemay et al., 2021). Furthermore, the synthesis or release of compounds that either have an antimicrobial effect or act as nutrients for the bacteria may vary between the different parts of the blade. This was described for phenolic substances in the kelp *L. hyperborea* and likely contributed to differences in the microbial composition (Bengtsson et al., 2012).

We found a higher proportion of *Planctomycetes* at the apex and *Actinobacteriota* almost exclusively in the meristem. Both phyla are found in brown macroalgae (Hollants et al., 2013) and on *S. latissima* [apex: (Staufenberger et al., 2008); meristem: (Tourneroche et al., 2020)]. *Planctomycetes* contain many sulfatase genes (Wegner et al., 2013), which help degrade sulfated polysaccharides. They may also be involved in degrading polysaccharides from the extracellular matrix of microbial biofilms (Parrot et al., 2019), which may explain their higher relative abundance in older tissues that exhibit first signs of degradation. *Actinobacteriota*, on the other hand, is a diverse phylum that has successfully colonized a wide range of habitats (Ul-Hassan and Wellington, 2009), but we currently do not know what features make them successful colonizers of the *S. latissima* meristem.

The bacterial core also reveals the shift from a low to a higher diversity as the blade ages, with four genera found in both algal parts and five additional genera found in >90% of apex samples. Those taxa were also found on the meristem part of *L. digitata* (Ihua et al., 2020) and on the blade of *L. setchellii* (Lemay et al., 2021), *Taonia atomaria* (Paix et al., 2021), and *U. lactuca* (Comba González et al., 2021). Our results are furthermore consistent with the core microbiota found in *S. latissima* and *L. hyperborea* in the United Kingdom (King et al., 2022). *Granulosicoccus* sp. (*Gammaproteobacteria*) was one of the most abundant genera (12% of total reads) and included several ASVs overexpressed in the meristem samples. This genus was also found abundantly on the youngest parts of the sister species *S. japonica* (Balakirev et al., 2012; Zhang et al., 2020) and other kelps like *L. setchellii* (Lemay et al., 2021)*, L. hyperborea* (Bengtsson et al., 2012)*, Macrocystis pyrifera*, and *Nereocystis luetkeana* (Weigel and Pfister, 2019; Ramírez-Puebla et al., 2022), reinforcing the idea of a strong association between *Granulosicoccus* and the kelp tissue. This genus might help its host by providing vitamins (vitamin B12, for example) and reduced nitrogen (Kang et al., 2018; Capistrant-Fossa et al., 2021; Weigel et al., 2022). In the same vein, the genus *Algitalea* (*Flavobacteriaceae*; 3% of total reads) was one of the “apex” bacterial core genera. This genus belonged to the pioneer bacterial communities found on the apical parts of *T. atomaria* (Paix et al., 2020). Also, the *Flavobacterium* lineage has been recognized as necessary in the decomposition processes of organic matter during algae blooms (Riemann et al., 2000; Pinhassi et al., 2004) and thus may participate in the decay process occurring at the algal apices.

### 4.2. Regional specificities: tides, seawater, and genetic background

Aside from the apex/meristem duality, the region of origin also was a decisive separation factor. (Lachnit et al., 2009) have already shown this region-dependent separation by comparing global epibacterial communities of algae from the North and Baltic Seas. In their study, only *S. latissima* showed regional differences within conspecific algae (contrary to the two other studied *Phaeophyceae*). At a larger geographical scale, results on *Ulva* sp. and *Agarophyton vermiculosum* (Roth-Schulze et al., 2018; Bonthond et al., 2020) suggested that the seaweed microbiota composition, diversity, and functions strongly depend on the local scale, but also shows that processes are acting at larger scales to shape this microbial community, and they need to be identified.

In our study, the regional differences were more pronounced than the seasonal differences obtained for one sampling site, suggesting that region and not just variability between sampling dates was the driving factor. The regional differences might be due to several abiotic factors. For instance, the tidal ranges for the three regions decrease going north: 10 meters in Roscoff, 3 meters in Helgoland, and less than 1 meter in Norway. In the same vein, winter seawater temperatures are lower in the north, which might favor psychrophilic strains over mesophilic communities, as found in a culture-based study on the surface bacteria of *L. longicruris* (Laycock, 1974). Also, increasing time exposure to rain, wind, or sunlight (UV), waves, currents, hydrostatic pressure, pH, and salinity can lead to cellular stress and, by extension, senescence. This changing environment can offer new ecological niches for different bacteria. Lastly, bacterial communities can be altered by nutrient supply, interspecies competition, and viral infection (Fuhrman et al., 2015; Stal and Cretoiu, 2016).

However, the regional differences might also be due, in part, to the algal genotype. Using single nucleotide polymorphisms (SNPs) and microsatellites, Guzinski et al. (2016, 2020) determined that *S. latissima* individuals from Brittany, Helgoland, and Norway are genetically distinct, and this might lead to the attraction of different bacterial species (Griffiths et al., 2019). Furthermore, *S. latissima* displays a unique lipidomic signature depending on its geographic origin (Monteiro et al., 2020), as the content of chemical elements (C, H, N, S), fatty acids, and lipids varies depending on the region. These molecules are common components of membranes (Harwood, 2004) and might influence the attractiveness of the algal surface for several bacterial strains. Lastly, variability, as described by Zhu et al. (2021), was not monitored in this study but could be included in future sampling schemes to elucidate the relationships between algal morphology and microbiome or vice versa.

### 4.3. Shifts in bacterial communities depending on the season

The third factor we examined was seasonality. We also provided the available abiotic data from a nearby monitoring site to discuss possible environmental drivers of the seasonality, but please note that our dataset comprising only four time points is insufficient to establish statistically meaningful correlations. Laycock (1974) observed that as the seasonal temperatures decreased, the bacterial communities of *L. longicruris* shifted from mesophilic to psychrophilic strains. When working with *L. hyperborea,* Bengtsson et al. (2010) hypothesized that the seasonal succession in the bacterial communities might be explained by abiotic factors like seawater temperature and biotic factors such as seasonal changes in the kelp substrate. Indeed, seawater temperature alone does not seem to have been the most important factor in our data, as the seawater was coldest in winter and spring (<12°C), but the samples from autumn and winter were more similar in their bacterial communities. Other physicochemical parameters might also play a role. Nitrogen is an important element for organisms, and microorganisms can take up nitrogen in different forms such as nitrate, nitrite, ammonium, urea, organic nitrogen, and in some cases, dinitrogen gas (N2), depending on the organism (Zehr and Ward, 2002). Nitrate, nitrite, and ammonium concentrations follow seasonal variations, with nitrates being lower in summer and higher in winter and nitrites at their highest in autumn. Phosphorus is another essential nutrient for primary production in the euphotic zone. Most of the phosphorus is present in the oxidized form as free phosphate or bound to organic matter. The phosphate concentration was lowest in springtime. Several ASVs overrepresented in spring belong to the *Roseobacter* clade, and Atlantic strains of this genus are known to possess abundant high-affinity phosphorus uptake systems, constituting likely adaptations to low environmental phosphate concentrations (Newton et al., 2010).

Lastly, seasonal variations may also be due to seasonal changes in the alga’s chemical composition. For instance, Schiener et al. (2015) demonstrated that in *S. latissima*, polyphenol levels are higher between May and July and then decrease, reaching their lowest in March. This could be interpreted as a defense against bacterial colonization as the seawater temperature rises, polyphenols being known for their wide range of antimicrobial properties (Zhang et al., 2006; Daglia, 2012). Also, carbohydrate content (laminarin and mannitol) is higher in summer (Schiener et al., 2015), and these are both substrates easy to degrade by the bacteria (Alderkamp et al., 2007; Jeske et al., 2013; Groisillier et al., 2015). Similarly, algal iodine content is generally lower in summer (Nitschke et al., 2018), and the algae’s production of toxic iodine compounds may control the surface biofilm and repulse microbial pathogens (Rodeheaver et al., 1982; Gobet et al., 2017). Regardless of the mechanisms, please note that seasonal changes may vary from one site to another, and this has also been previously shown to impact the seasonal changes in the microbial communities associated with *Macrocystis pyrifera* (Florez et al., 2019). Therefore, any conclusions drawn here about seasonality are valid only for the Roscoff site.

### 4.4. Is there a microbial signature characteristic for algae in poor health?

Some bacteria affect the alga in a deleterious manner by decomposing cell material, like alginate and laminarin (Laycock, 1974; Dimitrieva and Dimitriev, 1997; Sawabe et al., 1998b; Ivanova et al., 2003) or by causing diseases like *Alteromonas* species (Vairappan et al., 2001; Peng and Li, 2013) and species of *Pseudoalteromonas* (Sawabe et al., 1998a). Ihua et al. (2019) have shown that the microbial communities (phyla level) associated with intact *Ascophyllum* differ from rotting algae, suggesting that the decay process might shape the associated bacterial community. Similarly, the microbial communities of *Ecklonia* are strongly associated with the algal condition (stressed or not) more than with other variables (Marzinelli et al., 2015). Moreover, the core bacterial community characteristic of healthy algae may be lost when hosts are subjected to stress, and the microbiota of stressed individuals of *Ecklonia* were more similar to each other at a given location than those on healthy hosts (Marzinelli et al., 2015); which is contrary to the so-called Anna Karenina principle (Zaneveld et al., 2017; Ma, 2020), stating that all “healthy” microbiomes are alike and each “symptom” microbiome is “sick” in its own way. Lastly, in the red alga *Delisea pulchra*, certain bacteria have been shown to mitigate bleaching disease, likely by preventing dysbiosis (Li et al., 2022). If such effects are also common in *S. latissima*, and if they frequently involve the same bacteria, such bacteria could be statistically overrepresented in algae without symptoms.

In our study, the changes in bacterial communities between healthy and diseased individuals are visible only at the ASV level, and we found 9 ASVs that were differentially expressed between the healthy (5 ASVs) and diseased samples (4 ASVs). ASVs characteristic for the latter belong to the genus *Tenacibaculum* and the *Alteromonadales*, known for their alginate lyase activities (Thomas et al., 2019), and to the *Roseobacter* clade, known for their production of quorum-sensing molecules, a phenomenon involved in virulence and pathogenicity (Buchan et al., 2005; Wagner-Döbler and Biebl, 2006; Brinkhoff et al., 2008). One of the healthy specific ASVs is a *Granulosicoccus* sp., emphasizing the importance of this genus in the algal microbiota. The fact that several ASVs were found to differ indicates that, regardless of the type of disease, an alga that is not well will undergo characteristic changes in the microbiome. Moreover, these ASVs signatures are probably stable because they are derived from different places, times of the year, and symptoms, as shown for *Ecklonia* (Marzinelli et al., 2015). Although this would require additional developments, these signatures might also be helpful as bioindicators for kelp health.

## 5. Conclusion

In conclusion, our study provides an extensive overview of the *S. latissima* microbiome and highlights several factors driving its variability. In particular, the observation that the blade part had a more profound impact on the microbial composition than region or season underlines the extent to which algal hosts select their associated microbiota. Our discovery of microbial signatures characteristic for diseased *S. latissima* individuals that persist in our dataset independently of the disease symptoms further supports this hypothesis. Given the variety of symptoms observed in our samples, it is unlikely that the same bacteria could be the causative agents in all cases. Rather, the different types of disease likely cause similar changes in the host, which would lead to similar microbial changes. Understanding these signatures will be of interest for fundamental research on the different algal diseases, and may lead to the development of molecular markers of host health to survey natural populations or aquacultures.

## Data availability statement

The datasets presented in this study can be found in online repositories. The names of the repository/repositories and accession number (s) can be found at: https://www.ebi.ac.uk/ena, PRJEB47035.

## Author contributions

BB-D and SD: study design. BB-D, SF, and SD: sampling. B-D, EL, and GT: experiments performance. BB-D and SD: data analysis and wrote the manuscript. CB: provide valuable input and corrected the manuscript. All authors contributed to the article and approved the submitted version.

## Funding

This work was funded partially by ANR project IDEALG (ANR-10-BTBR-04) “Investissements d’Avenir, Biotechnologies-Bioressources,” the CNRS momentum call (2017), and by the European Union’s Horizon 2020 research and innovation program under grant agreement No 730984, ASSEMBLE Plus project. BBD was funded by a joint Ph.D. scholarship from the Brittany region (Project HOSALA) and Sorbonne University (ED227).

## Conflict of interest

The authors declare that the research was conducted in the absence of any commercial or financial relationships that could be construed as a potential conflict of interest.

## Publisher’s note

All claims expressed in this article are solely those of the authors and do not necessarily represent those of their affiliated organizations, or those of the publisher, the editors and the reviewers. Any product that may be evaluated in this article, or claim that may be made by its manufacturer, is not guaranteed or endorsed by the publisher.

## Footnotes

^1^ https://www.somlit.fr/mysomlit/

^2^ https://benjjneb.github.io/dada2/tutorial.html

## Supplementary material

SUPPLEMENTARY FIGURE S1 Seasonal variations in (A) temperature, (B) salinity and (C) ammonium, (D) nitrites, (E) nitrates, and (F) phosphate concentrations. Roscoff, 2019. Legend for each month, rectangle: region inside 1st and 3rd quartiles, bold line: median value, dashed error bars: 1st and 9th deciles, red point: value for the selected year.

SUPPLEMENTARY TABLE S1 Taxonomic affiliations of over-expressed ASVs for each comparison (algal part, regions, seasons, and symptoms).
