# Supplementary figures and images for: The Saccharina latissima microbiome: Effects of region, season, and physiology
Source: Front Microbiol. 2023 Jan 6;13:1050939. doi: 10.3389/fmicb.2022.1050939 (PMC9858215; doi:10.3389/fmicb.2022.1050939)

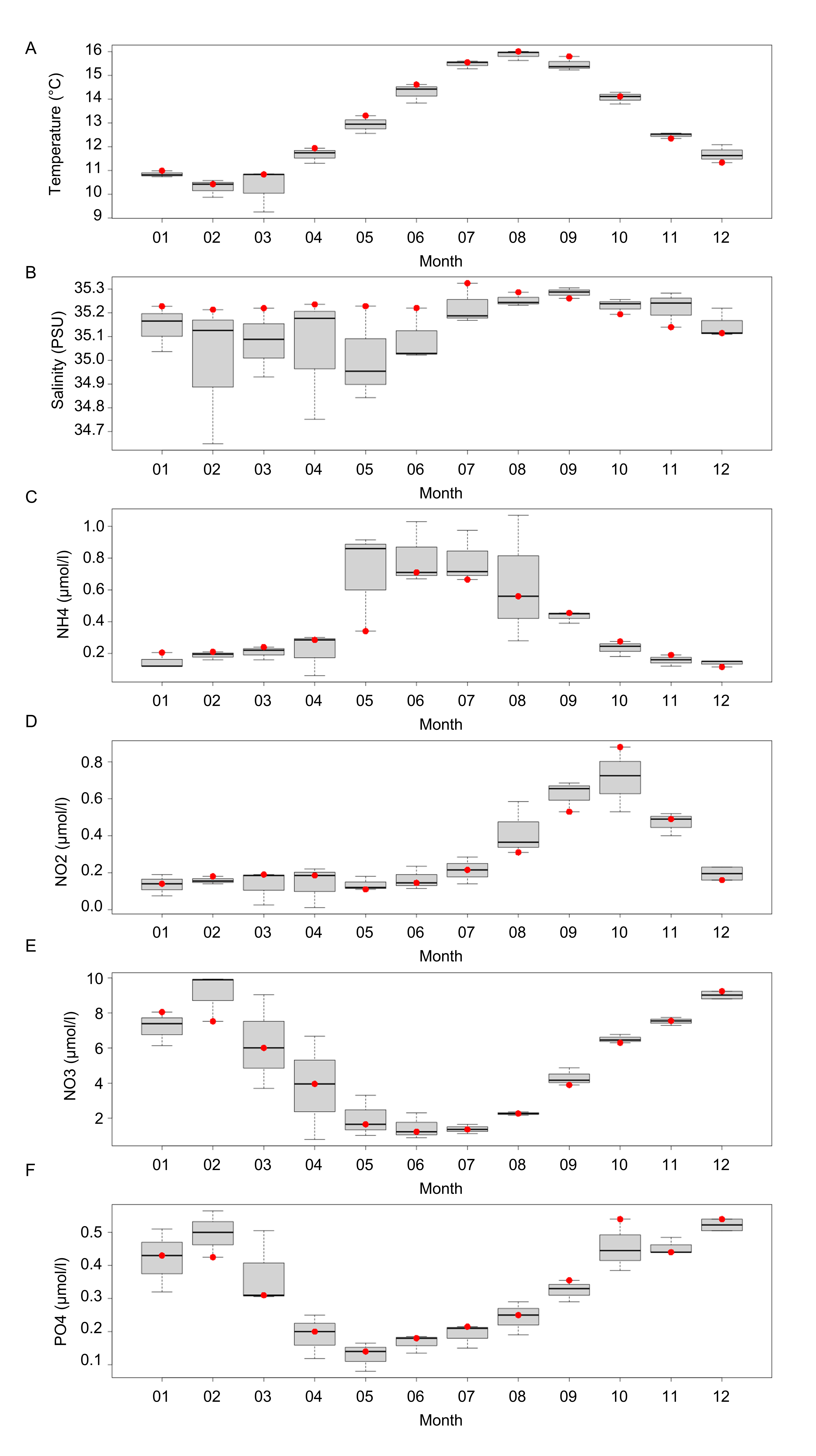

Supplement: SUPPLEMENTARY FIGURE S1 — Seasonal variations in (A) temperature, (B) salinity and (C) ammonium, (D) nitrites, (E) nitrates, and (F) phosphate concentrations. Roscoff, 2019. Legend for each month, rectangle: region inside 1st and 3rd quartiles, bold line: median value, dashed error bars: 1st and 9th deciles, red point: value for the selected year. [file Image_1.TIF]
